# Patterns of Objective and Subjective Burden of Informal Caregivers in Multiple Sclerosis

**DOI:** 10.1155/2015/648415

**Published:** 2015-05-20

**Authors:** E. Bayen, C. Papeix, P. Pradat-Diehl, C. Lubetzki, M. E. Joël

**Affiliations:** ^1^Department of Neuro-Rehabilitation, Pitié-Salpêtrière Hospital, 75013 Paris, France; ^2^Health Economics Department, LEDa-LEGOS, PSL, Paris-Dauphine University, 75016 Paris, France; ^3^Department of Neurology, Pitié-Salpêtrière Hospital, 75013 Paris, France; ^4^ER6 Unit, Pierre and Marie Curie University, Pitié-Salpêtrière Hospital, 75013 Paris, France; ^5^CRICM, Inserm UMR S975, Pierre and Marie Curie University, Pitié-Salpêtrière Hospital, 75013 Paris, France

## Abstract

*Background*. Home care for patients with Multiple Sclerosis (MS) relies largely on informal caregivers (ICs). *Methods*. We assessed ICs objective burden (Resource Utilization in Dementia measuring informal care time (ICT)) and ICs subjective burden (Zarit Burden Inventory (ZBI)). *Results*. ICs (*N* = 99) were spouses (70%), mean age 52 years, assisting disabled patients with a mean EDSS (Expanded Disability Status Scale) of 5.5, with executive dysfunction (mean DEX (Dysexecutive questionnaire) of 25) and a duration of MS ranging from 1 to 44 years. Objective burden was high (mean ICT = 6.5 hours/day), mostly consisting of supervision time. Subjective burden was moderate (mean ZBI = 27.3). Multivariate analyses showed that both burdens were positively correlated with higher levels of EDSS and DEX, whereas coresidency and IC's female gender correlated with objective burden only and IC's poor mental health status with subjective burden only. When considering MS aggressiveness, it appeared that both burdens were not correlated with a higher duration of MS but rather increased for patients with severe and early dysexecutive function and for patients classified as fast progressors according to the Multiple Sclerosis Severity Score. *Conclusion*. Evaluation of MS disability course and IC's personal situation is crucial to understand the burden process and to implement adequate interventions in MS.

## 1. Introduction

Living with a multiple sclerosis (MS) often leads to increasing disability and reliance on home care which is provided by an informal caregiver in 80% of cases [1]. Informal caregivers (ICs), that is, a nonprofessional person assisting a disabled person in activities of daily living [2], constitute a long-term free labour force for home care, avoiding costly institutionalization and publicly funded formal paid care of patients [3]. IC workload has long been considered invisible in MS [4] but growing economic constraints have generated health policymakers' and health professionals' interest in understanding the burden process in order to avoid IC risk of burnout or withdrawal from the caregiving role [5].

Although IC burden has been a key concept for decades, the definition [6] and ways of measuring it [7] vary widely. In this study we combined two dimensions of IC burden by referring to a theoretical background [6] hypothesizing that they would provide complementary information. First* objective* burden refers to patient's care needs associated with observable IC caregiving tasks [6]. The presence of objective burden thus points out that formal support inadequately substitutes for informal support. Second,* subjective* burden refers to feelings aroused in caregivers as they fulfill their caregiving functions [6]. Understanding of subjective burden allows intervention programs to be provided to alleviate IC burden. Additionally, understanding the burden process means identifying relevant determinants [8] and also considering MS disability severity course as MS is a chronic disease with various patterns of evolution. New approaches that are focusing on MS disability severity course (i.e., combining impairment over duration) are gaining popularity in translational research [9–12] but have not been yet translated to benefits for the management of home care needs. Therefore we hypothesized that the use of the Multiple Sclerosis Severity Score [9] to identify patients with fast progressive MS might shed new light on the burden issue in MS.

The present study is a prospective investigation of home-dwelling patients with MS and their ICs included in a French medical setting. The aims were to analyze (1) objective and subjective burden and (2) their determinants and (3) to describe burden patterns in the light of the MS disability severity course.

## 2. Methods

### 2.1. Design of the Cross-Sectional Study

The sample of patient-caregiver pairs was recruited by contacting MS patients who were consecutively seen in the Day Hospital of the Neurological Department of the Pitié-Salpêtrière Hospital (Paris, France) between March and October 2012. Inclusion criteria were home-dwelling patients with MS, having at least one primary IC, with no relapse at the time of the inclusion and during the last three months. The* primary* IC was defined as the family member or friend who was most responsible for day-to-day decision making and/or care of the patient. The inclusion form was filled in by a trained medical doctor who also gave the patient a written questionnaire to be completed by the primary IC and a stamped-addressed envelope.

### 2.2. Patient Assessment

Current level of disability was evaluated using the Expanded Disability Status Scale (EDSS), the Dysexecutive questionnaire (DEX), and bladder dysfunction. The EDSS is the most validated and widely accepted measure of disability in MS. Scores are assigned in half-point increments, ranging from 0 (normal neurologic exam) to 10 (death due to MS) with a primary focus on motor impairment; the first levels from 1.0 to 4.5 refer to people with a high degree of ambulatory ability and the subsequent levels from 5.0 to 9.5 refer to a gradual loss of ambulatory ability [14]. The DEX (IC assessment) is a 20-item questionnaire which measures changes in everyday life as a result of dysexecutive function, ranging from 0 (no problem) to 80 (higher impairments) [15]. Bladder dysfunction (including the use of intermittent catheterisation) was assessed through a dichotomised score (known overactive bladder and/or voiding dysfunction or not). Socioeconomic variables entailed whether the patient had some professional activity and whether the patient received outpatient rehabilitation and formal care. Mean daily formal care time (FCT) was calculated from the number of publicly funded formal care hours per typical care day and the number of days per typical care week. The Multiple Sclerosis Severity Score (MSSS) [9, 13] was used to sort patients by MS progression course over time (scatter plot figures). The MSSS was developed using large databases (*n* = 9892 patients with a MS duration ranging from 1 to 30 years) and uses a probabilistic method to assign a disease severity score adjusted for disease duration (relating a patient's EDSS to the distribution of disability in patients with the same MS duration). The MSSS table was used in the perspective of the disability severity course, whatever the history of relapsing-remitting or progressive form is; throughout the paper, the terms “fast progressors” and “slow progressors” [16] were used to denote patients with a respective rapid and slow disability course at the time point of the study (identified in the matrix of distribution of the MSSS deciles as patients over the 8th decile and under the 2nd decile, resp.).

### 2.3. Primary Informal Caregiver Assessment

The self-report Medical Outcome Study Short Form-12 [17] (SF12v1) was used to evaluate the health status of ICs with calculation of two summary measures (Physical Component Summary (PCS) and Mental Component Summary (MCS)) which are standardized to reflect a general population mean of 50 with a standard deviation of 10. Objective burden was assessed with the Resource Utilization in Dementia-Part 1 [18] which evaluates informal care time (ICT) in three different categories: Activities of Daily Living (ADL: toilet visits, eating, dressing, grooming, walking, and bathing); Instrumental Activities of Daily Living (IADL: shopping, food preparation, housekeeping, laundry, transportation, taking medication, and managing financial matters); Supervision Time (ST: average time spent preventing a dangerous event). The ICs were asked whether the patient needed help in each of the three categories and consequently to state for how many hours per day and for how many days in the last month they had assisted the patient. These scores yielded a mean daily ICT, ICT-ADL, ICT-IADL, and ICT-ST. Subjective burden was estimated with the Zarit Burden Inventory [19] (ZBI: 22 questions rated on a 5-point scale ranging from no burden = 0 to overburdened = 4). Clinical cut-off scores graded ZBI severity as mild (range 0–20), mild to moderate (21–40), moderate to severe (41–60), and severe (61–88) [20]. Socioeconomic variables were recorded by asking whether the informal care workload had been impacting the IC professional, leisure, and social network activity, respectively. The conflicting role variable was coded dichotomously by attributing a score of 1 if the IC was working and/or if he/she was in charge of an elderly person or a child.

### 2.4. Statistical Analyses

The patients' and caregivers' characteristics and scores were described by means, standard deviations (SD), and ranges (minimum–maximum) for continuous variables and counts and percentages for categorical variables. In the case of missing values, percentages were calculated and the denominator was specified. Inclusion data of the patients whose IC returned the questionnaire were compared with those whose IC did not return the questionnaire using Khi2 and Mann-Whitney* U* tests. SF12 MCS-PCS scores for primary informal caregivers were compared with global norms (men and women older than 18 years) of a representative sample of the French population [2] using* t-*tests. Correlation coefficient tests (coefficients reported on scatter plot figures) were used to evaluate the univariate association between the ICs' outcomes (ICT and ZBI) and patient and caregiver variables (*α* error set at 5%). Two linear regression models were computed with ICT and ZBI as dependent variables. For both predictive models, three blocks of independent explanatory variables were selected based on previously published studies of burden in MS [4, 8, 21, 22], on univariate analyses, and on screening for multicolinearity as follows: (1) patient's variables (EDSS, DEX, urinary intermittent catheterisation, and MS duration); (2) caregiver's variables (gender, coresidency, conflicting role, SF12-MCS, and SF12-PCS); (3) socioeconomic variable (formal care support at home). For each multivariate model an ascending strategy was computed with a progressive addition of each block of variables. The Akaike Information Criterion (AIC) which enables to select the most parsimonious model when dealing with empirical data [23] was performed during this stepwise procedure to verify the contribution of the three successive blocks to the full model. A logistic regression model was computed with FCT as a dichotomous dependent variable and the same above-mentioned independent variables in order to compare formal and informal care determinants. A logistic model was used because the FCT variable had a value of zero in more than half of the cases. Statistical analyses were performed using STATA v12.1.

### 2.5. Ethical Concerns

In accordance with French legislation, the study was approved by the French Ethical Research Committee (Comité de Protection des Personnes Ile de France VI) and declared to the French National Commission for Data Protection and Liberties (Commission Nationale de l'Informatique et des Libertés). In accordance with French legislation, patients and informal caregivers were informed about the purpose of this study upon inclusion in the database and consent was obtained after oral and written information.

## 3. Results

### 3.1. Patients and Primary Informal Caregivers

A total of 216 patients were included, of which 99 primary IC returned the self-administered postal questionnaire. There were no significant differences between the inclusion data (age, gender, and EDSS; all *p* > 0.05) of the patients whose IC returned the questionnaire and those whose IC did not return the questionnaire.

The patients included were mostly women (63%) with a mean age of 46 years and a wide range of EDSS scores (from 1.0 to 9.5) (Table 1). A significant level of cognitive impairment was found with the most frequent dysexecutive disorders (DEX) reported in order as follows: apathy and lack of drive, aggression, planning problems, distractibility, poor decision-making ability, euphoria, shallowing of affective responses, and inability to inhibit responses. Twenty fast progressors and no slow progressors were identified. The fast progressors had a mean EDSS of 7.3 (range = 4–9.5), DEX of 25 (range = 0–63), and MS duration of 9.7 (range = 2–22 years) and 39% used intermittent catheterisation. ICs caring for fast progressors had a high mean ICT of 9.6 hours/day and had a mean ZBI of 36.

The primary IC was almost exclusively a family member, living with the patient (83%), with a high proportion of spouses (70%) (Table 1). IC health status was significantly lower than that of the general French population [2] for both mental and physical components of the SF12 (*p* < 0.001). Eighteen percent of ICs had adjusted their professional activity and at least one-third had experienced an impact on their personal and social lives.

### 3.2. Objective Informal Care Burden

**Figure 1:**
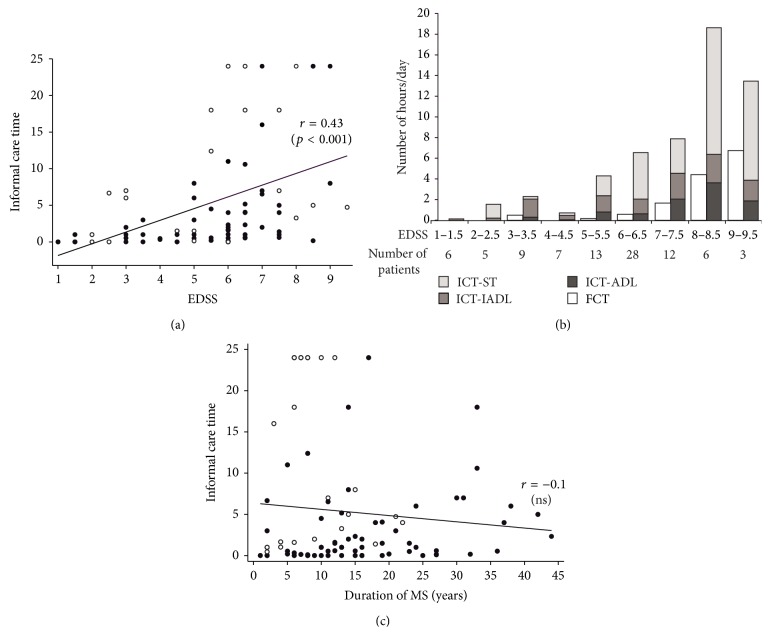
Informal caregiver objective burden (informal care time (ICT)): (a) scatterplots of the correlation between EDSS score and ICT. White dots signify that patient's Dysexecutive questionnaire score is over 75th percentile (otherwise dark dots), (b) ICT-sub activities and formal care time (FCT) as a function of EDSS. ADL = Activities of Daily Living; IADL = Instrumental Activities of Daily Living; ST = Supervision Time, and (c) scatterplots of the correlation between MS duration and ICT. White dots specify fast progressors (i.e., patients with a rapid disability course identified in the Multiple Sclerosis Severity Score [9, 13] table over the 8th decile) and dark dots specify otherwise.

The proportion of use of home care for patients was distributed as follows: 20% (19/96) patients received no informal care (ICT = 0) and 80% (77/96) received informal care. Among these 77 patients, 28 (36% of patients receiving informal care) received mixed care (i.e., a combination of formal and informal care) and 49 (64%) received informal care only. No patient received only formal care. The level of objective burden was high with a mean ICT of 6.5 hours/day whereas mean FCT amounted only 2.9 hours/day (Tables 1 and 2).  Figure 1(a) shows that there was a wide range of ICT (from 0 to 24 hours/day) and that there was a linear increase in ICT with higher EDSS. As shown in Figure 1(b), FCT might partially substitute or complement ICT only for patients with higher EDSS scores (mean FCT amounting to 50% of mean ICT for patients with EDSS scores of 9 and 9.5). Figure 1(b) shows that Supervision Time (ICT-ST) made up a major part of ICT. Two populations of patients can be distinguished in Figure 1(c) (fast progressors represented by white dots and others), with higher ICT values for fast progressors. The figure also shows that ICT as a function of MS duration is stable when data from all the patients are plotted together (regardless of the disability course of the MS). It must be noted that the stable fit is due to the fact that ICT for fast progressors (represented by white dots) is high early after the disease onset.

### 3.3. Subjective Informal Care Burden

**Figure 2:**
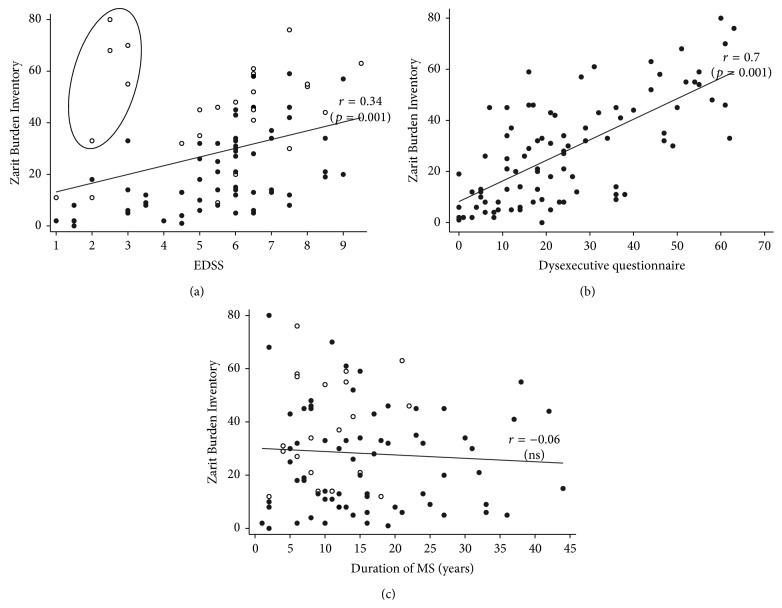
Informal caregiver subjective burden (Zarit Burden Inventory (ZBI)): (a) scatterplots of the correlation between EDSS scores and ZBI scores. White dots signify that patient's Dysexecutive questionnaire score is over 75th percentile (otherwise dark dots); circle represents patients with EDSS score under 4 and Dysexecutive questionnaire score over the 85th percentile, (b) scatterplots of the correlation between Dysexecutive questionnaire scores and ZBI scores, and (c) scatterplots of the correlation between MS duration and ZBI. White dots specify fast progressors (i.e., patients with a rapid disability course identified in the Multiple Sclerosis Severity Score [9, 13] table over the 8th decile) and dark dots specify otherwise.

Objective and subjective burden scores were correlated (Spearman rho = 0.54, *p* < 0.001). The assessment of subjective burden (ZBI) showed that more than half of ICs (54%) reported perceived burden (Table 3). The most frequent ZBI items reported were in order fear for their relative's future, stress because of caring while also facing other professional/familial responsibilities, feeling embarrassed about their relative's behaviour, not having enough time for themselves, feeling that they should be doing more for their relative, and feeling burdened with caring for their relative.  Figure 2(a) shows an increase in subjective burden associated with higher patient EDSS.  Figure 2(b) shows that higher subjective burden was strongly and significantly associated with a higher level of patient DEX.  Figure 2(c) shows that ZBI score as a function of MS duration is stable when data from all the patients are plotted together, with the scores of the fast progressors pulling the fit upwards in the early stages of MS.

### 3.4. Determinants of Objective and Subjective Burden and of Formal Care Provision

The two multivariate regression models showed that a high level of global disability (EDSS) and dysexecutive disorders (DEX) were significantly positively associated with both ICT and ZBI outcomes (Table 3). In addition, several caregiver-related factors were positively associated with ICT (female gender and coresidency) and negatively associated with ZBI scores (SF12-MCS). However, the logistic regression model showed that only global disability (EDSS) was a significant determinant of FCT. For the ZBI and the ICT outcomes, the three blocks of variables included in the full model resulted in a better goodness of fit as indicated by a better (lower) AIC than the initial model (including patient's variables only).

## 4. Discussion

In this prospective cross-sectional study of 99 patients with MS (included from EDSS = 1 to EDSS = 9.5) from a major referral hospital in France, IC burden was found noticeable, both for its objective and its subjective dimensions. A major finding was that both burdens were found high rapidly after the onset of the disease when patients showed early and severe cognitive profiles, and when patients were classified as MS fast progressors according to the Multiple Sclerosis Severity Score. Accordingly, both types of burden were predicted by similar patient's core clinical outcomes (global disability and cognitive-behavioral impairment). However, distinct predictors were identified for each type of burden, such as coresidency and IC's female gender for objective burden and IC's poor mental health status for subjective burden. This multidimensional analysis of burden might therefore help to best address patient's and caregiver's needs in MS.

The present study confirms that being an informal caregiver is associated with poor mental and physical health outcomes (as assessed by the SF12) and with personal sacrifices (as assessed by withdraw from work, leisure, and social network) [8, 24]. The moderate level of subjective burden was similar to another study in MS which found a ZBI score of 22 (*n* = 278 and mean EDSS = 4.4) [21]. The level of objective burden was high and concordant with several previous estimations of total ICT for MS patients ranging from 4.6 to 12 hours/day for moderately and severely disabled patients, respectively, (Belgium) [25] to 11.5 hours/day (Spain) [26] or to over 20 hours/week (United States) [22]. Interestingly, the subactivity with the highest value of ICT was Supervision Time. This is known to be a major component of ICT for adults with neurological disability [27] and is required when patients are at risk of harming themselves because of either physical or cognitive loss of autonomy. This large amount of informal caregiving time found here raises concerns because it is unpaid and is frequently delivered by working-aged IC. A significant proportion of IC reduced their working hours and their leisure and social activities what shows the short-term socioeconomic impact of hours of care commitments. In contrast, the provision of home care by the state found here was low compared with informal care and compared with home-based rehabilitation services. As shown on the histogram a “crowding-out effect” on formal care was found for the most severe EDSS scores only. This might suggest that French state delays the substitution of informal care by formal paid care until care obligations cannot be met anymore because patients' disability is too severe. These findings raise concern regarding the lack of proper financial compensation for ICT. In France, a modest newly introduced indemnity for ICT compensation (€3.62–€5.43 per hour of informal care) in the form of the disability compensation benefit is a first step toward an official recognition and support of IC's involvement [28]. Further financial propositions for indirect forms of compensation (tax relief and vouchers for respite) could also be appropriate alternatives to maintain IC continued involvement in the caregiving relationship.

The results of the bidimensional and multivariate tests showed that global disability (EDSS) and cognitive impairment (DEX score) were significant simultaneous determinants of both objective and subjective IC burden. This confirms that the EDSS is an explanatory factor of burden [29] and is meaningful since the EDSS is the principal outcome measure used in routine clinical monitoring of patients with MS. The present study demonstrates the major impact of the patient's dysexecutive function on caregivers, which has been less documented than the role of the patient's physical impairment [30]. This is all the most relevant as cognitive disorders have been shown to be largely underestimated clinically in MS [31]. Therefore, systematic early cognitive-behavioural screening is needed in MS in order to propose adapted therapies to patients [32] and intervention programs for ICs to inform them regarding cognitive-behavioural impairment and coping strategies [33].

A major result of this study is that high levels of both types of burden occur in part from the very onset of MS, what appears counterintuitive at first sight given the fact that MS is a chronic disease with an average long-term worsening of patients' state over decades. No clear pattern of a gradual increase in objective and subjective burden over time emerged in this cross-sectional analysis of a day hospital sample of MS patients. In the early stages of MS, a high level of burden might be explained by the frequency/severity of the relapses [34] but also by a rapid progression course of the global disability and by severe cognitive profiles, as shown in the present study. It seems therefore crucial to consider the disability severity course (i.e., combining impairment and duration) [9, 12, 13] through the use of the MSSS table for instance and not simply the duration of the disease when monitoring the burden process. Early tracking of MS aggressiveness should be carried out in order to make patients with such profiles quickly eligible for publicly funded formal care because they require much care time and are a source of stress for ICs. The perception which the IC has of the unpredictable disease course and prognosis might also account for the early onset of subjective burden [35]. Conversely, both types of burden can be decreased over time by better home health care organization (additional caregivers and substitution by formal care) [36] or a new life project. Finally, positive caregiving experiences and a sensation of fulfilment may also result in alleviating IC subjective burden over time [37].

The multivariate predictive models showed that subjective and objective burden were determined by different cross-sectional caregiver-related characteristics. Being a female caregiver was predictive of higher levels of objective burden, which confirms that the major role played by female caregivers in other neurological disorders [38, 39] is also true for MS, although contrasting results have also been reported [22]. Coresidency status was associated with an increase in objective burden, mostly because cohabiting ICs are available to provide help at any time. However, since sharing a household also means sharing domestic tasks, it may be difficult to distinguish between “normal” and “additional” (because of the disability and the neurological fatigability) IADL tasks. Also, supervision might actually be “part-time” because the IC performs multiple tasks whilst supervising the patient. These time overlaps are called “IADL-shared activities” and “ST-joined activities” and might lead to differences in the estimation of ICT as already suggested [40]. However, publicly funded formal care in France is primarily allocated for personal care (ADL needs), as confirmed here by the logistic regression model of formal care being predicted by the EDSS variable only. Formal care provision by the state does not generally alleviate the IC workload regarding IADL and ST, despite the fact that the lack of participation in these subactivities is related to neurological fatigability and cognitive disorders and is producing a major distress in caregiver's lives [41]. Finally, the impact of poor IC mental health status on their experience of subjective burden confirms the need for support of MS caregivers [42]. As a whole, public health professionals should encourage direct intervention programs targeting ICs [43, 44]; more of them should be implemented in MS to support ICs with cognitive-behavioral therapy and skill building, personal coping strategies, and peer-support and social support in general [45].

A first strength of this work lies in the use of both an objective and a subjective burden measure. In particular, the objective burden measure improves the comprehensiveness of home care needs in MS and of the insufficient crowding-out effect of formal care. It also provides robust arguments for an official and financial recognition of IC role in the future given the socioeconomic impact of IC care involvement. A second strength is to make an original use of the MSSS thereby demonstrating the utility of monitoring MS aggressiveness to improve home care management.

A first limitation of the study is that caregiver's personal comorbid disorders that existed prior to their care involvement were not documented although they might have impacted their physical and mental health as well. Second, caregiver's unwillingness to be replaced by professional caregivers was not assessed here but it has been shown that such a preference might in some cases contribute to a lower rate of formal care [46].

To conclude, early identification of fast progressors and patients with executive dysfunction is critical for the implementation of efficient formal care support. Understanding the multidimensional burden process and needs of ICs will help to propose adequate intervention programs and to facilitate their continued involvement.

## Figures and Tables

**Table 1:** Participant's characteristics and outcomes.

	Mean (SD; range [min–max]) Count (%)
*Patients* (*N* = 99)	
Demographic characteristics	
Gender (female)	59/92 (64%)
Age (years)	46.4 (13.9; [17–77])
Clinical outcomes	
MS subtype	
Relapsing-remitting	41 (41%)
Primary and secondary progressive	58 (59%)
Duration of MS (years)	14.8 (10.2; [1–44])
EDSS score	5.5 (2.0; [1–9.5])
DEX score	24.8 (17.9; [0–63])
Bladder dysfunction (yes)	68 (74%)
Urinary intermittent catheterisation (yes)	19 (19%)
Current disease-modifying treatment (yes)	76/91 (84%)
Socioeconomic variables	
Professional activity (yes)	45/94 (48%)
Outpatient rehabilitation (yes)	68 (69%)
Use of formal care (yes)	28 (28%)
Mean formal care time (hours per day)	2.9 (4.7; [0.3–24])
*Informal caregivers* (*N* = 99)	
Demographic characteristics	
Gender (male)	51 (51.5%)
Age	52.3 (14.4; [19–88])
Relationship to the patient	
Parent	21
Spouse	69
Brother or sister + friend	5 + 1
Child	3
Clinical outcomes	
SF12-PCS	41.6 (6.8; [10.5–55.2])
SF12-MCS	39.5 (11.0; [9.9–61.6])
Socioeconomic variables	
Coresident with the patient (yes)	82 (83%)
Professional activity (yes)	61 (61.6%)
Retired (yes)	27 (28%)
In charge of an elderly person + child <18 years (yes)	24 + 26 (51%)
Conflicting role (yes)	73 (74%)
Reduced professional activity due to caregiving (yes)	18 (18%)
Reduced leisure activities due to caregiving (yes)	52 (53%)
Reduced friend network due to caregiving (yes)	32 (32%)

**Table:** (a) Objective burden score (informal care time, *n* = 96)

	Patient-caregiver pairs (count)	Mean (SD; range [min–max]) Hours/day
Patient-caregiver pairs with no ICT	19	0
Patient-caregiver pairs with ICT^a^	77	6.5 (8.2; [0.07–24])
ICT tasks (subgroup)		
ICT-ADL	44	2.1 (1.7; [0.03–6])
ICT-IADL	76	1.79 (1.6; [0.06–6])
ICT-ST	34	9.6 (9.6; [0.33–24])

**Table:** (b) Subjective burden score (Zarit Burden Inventory, *n* = 96)

	Caregivers (count)	Mean (SD; range [min–max])
Caregivers' ZBI	96	27.3 (19.9; [0–80])
ZBI severity (subgroup)		
Mild	44	9.6 (5.7; [0–20])
Mild to moderate	25	30.0 (4.7; [21–37])
Moderate to severe	21	48.9 (6.1; [41–59])
Severe	6	69.7 (7.3; [61–80])

^a^Patients receiving help from an informal caregiver for ADL and/or IADL and/or ST.

Note: ICT: informal care time; ADL: Activities of Daily Living; IADL: Instrumental Activities of Daily Living; ST: Supervision Time; SD: standard deviation.

**Table 3:** Factors associated with informal care burden and with formal care provision.

Multivariate model (*n* = 81):	Linear regression		Logit
	Objective burden ICT	Subjective burden ZBI	Formal care support (Yes/no)
(1) Patient variables			
EDSS^a^	1.2 (0.002)	2 (0.02)	0.8 (0.004)
DEX^a^	0.1 (0.03)	0.6 (<0.001)	−0.004 (ns)
Urinary Intermittent catheterisation^b^	0.8 (ns)	3.9 (ns)	−0.9 (ns)
MS duration^a^	−0.13 (0.08)	−0.2 (ns)	0.02 (ns)

(2) Caregiver variables			
Gender^b^ (ref. male)	3.3 (0.03)	4.1 (ns)	−0.8 (ns)
Coresidency^b^	4.5 (0.01)	−2.5 (ns)	0.1 (ns)
Conflicting role^b^	−1.2 (ns)	1.6 (ns)	−0.2 (ns)
SF12-MCS^a^	−0.08 (ns)	−0.5 (<0.001)	−0.05 (ns)
SF12-PCS^a^	−0.2 (ns)	−0.3 (ns)	−0.06 (ns)

(3) Socioeconomic variable			
Formal care support at home^b^	0.1 (ns)	4.5 (ns)	—
*R* ^2^	0.39	0.73	
	AIC (block1) = 663	AIC (block1) = 533	
	AIC (block1-2) = 632	AIC (block1-2) = 519	
	AIC (block1-2-3) = 626	AIC (block1-2-3) = 505	
Pseudo-*R* ^2^			0.28

Note: results show estimates of unstandardized beta coefficients for the two linear full regression models and of log odds for the logit model; *p* values in brackets are specified when *p* < 0.1, and they are nonsignificant (ns) otherwise.

Interpretation: ^a^for continuous variables, for example, the expected change in ICT per 1-unit increment in EDSS is 1.2 hours and ^b^for dichotomic variables, for example, the expected change in ICT when the caregiver is a female is 3.3 hours.

Note: ICT = informal care time; ZBI = Zarit Burden Inventory; AIC = Akaike Information Criterion.
